# Optimized menu formulation to enhance nutritional goals: design of a mixed integer programming model for the workers’ food program in Brazil

**DOI:** 10.1186/s40795-023-00705-0

**Published:** 2023-03-20

**Authors:** Marina Padovan, Fernando Ribeiro de Senna, Juliana Klein Kimura, Samara Tortorella Nascimento, Antonio Carlos Moretti, Caroline Dário Capitani

**Affiliations:** 1 Multidisciplinary Food and Health Laboratory (LabMAS), School of Applied Sciences, State University of Campinas (UNICAMP), 1300 Pedro Zaccaria St, Jd Santa Luzia, Limeira, São Paulo 13484-350 Brazil; 2 Institute of Mathematics, Statistics and Scientific Computing (IMECC), University of Campinas (UNICAMP), University City, Campinas, São Paulo 13083-856 Brazil; 3 School of Applied Sciences, State University of Campinas, 1300 Pedro Zaccaria St, Jd Santa Luzia, Limeira, São Paulo 13484-350 Brazil

**Keywords:** Menu development, Mathematical modeling, Mixed integer programming, Workers’ health, Institutional foodservices

## Abstract

**Background:**

In Brazil, institutional foodservices are required to meet the recommendations of the Workers? Food Program (WFP), a national public policy used to plan collective menus. The current study aimed to propose a mathematical model to generate a one-month menu that meets the nutritional recommendations of the WFP, with low cost and good quality.

**Methods:**

We considered aspects related to the eating habits of the Brazilian population, spacing of repetitions between the dishes, texture combination, and monotonicity of colors of the dishes served. A mixed integer programming model was built to formulate daily menus for an institutional foodservice for one month. The menu consisted of a base dish, a base dish option, salads (2 options), a protein dish, a protein dish option, a side dish, and a dessert.

**Results:**

The model ensured compliance with the recommendations proposed by the WFP and the provision of healthy and nutritionally balanced meals. The menu generated met the recommendations of the WFP, with an average of 716.97 kcal/meal, including on average 58.28% carbohydrates, 17.89% proteins, and 24.88% total fats/meal.

**Conclusion:**

The model used can help in the menu elaboration dynamics of institutional foodservices, optimizing the work of the nutritionist in charge.

**Supplementary Information:**

The online version contains supplementary material available at 10.1186/s40795-023-00705-0.

## Background

Nutrient-based recommendations, often called Dietary Reference Intakes (DRIs), are commonly used for food intake assessment and planning [1–4]. However, when it comes to food for collectivities, specific recommendations are based on the Workers’ Food Program (WFP), a public policy established in 1976 [5] and revised in 2006 [6]. Collective eating includes workers, or diners, who usually eat one or more meals in the workplace in specific cafeterias or in the institutional foodservice of the company where they work, whether private or public, and in hospitals or schools. The guidelines described recommend balanced distribution of nutrients with the aim of providing fibers, sodium, and macronutrients in a balanced way to diners [6]. The national recommendations, based on the Dietary Guidelines for the Brazilian Population [4] also recommend that menus, in general, should be prepared prioritizing *in natura* and minimally processed foods. According to Veiros and Proença [7], a menu with good quality should offer harmonious color combinations and not frequently repeat certain culinary methods [8]. In the preparation of the menu in an institutional foodservice, it is necessary to consider the financial limits of the institution [9, 10] along with the adequacy of the nutritional recommendations provided for the WFP. Therefore, when organizing a menu, all these requirements should be addressed to meet national guidelines and promote healthy and affordable food choices [11].

### Problem description

Although the literature has no consensus on which methods should be adopted for the elaboration of menus served in an institutional foodservice, in practice the menus are prepared by technical managers (TM), usually trained nutritionists, focusing on meeting the demands of diners and striving for a healthy and balanced diet. However, these TMs, in most cases, must consider the contract, which often prioritizes costs and food preferences. Thus, the nutritionist can be overwhelmed by the task of thinking more broadly about the menu. That is, for its elaboration, aspects such as availability of labor, available equipment, food preferences, and number and types of culinary preparations contracted must be considered. Since cost management is an essential factor for organizing the activities of an institutional foodservice, the nutritionist often opts for less complex dishes, with lower cost and greater acceptability.

Menu planning can be performed manually, or with programs/softwares that use equivalent values (nutrients and quantities of ingredients, for example) and general aspects, thus lacking exclusivity to the demands of each diner. Given the numerous administrative tasks performed by the professionals, they frequently lack the time to analyze the nutrient composition of the menus or even quality aspects (combination of colors, textures, and flavors), and may not comply with the established regulations [4, 6]. As an example, studies developed in Brazil show non-compliance with national guidelines [12–14] and the frequent inclusion of ultra-processed foods, which are used for convenience or low cost [15].

Thus, considering all the demands on the TM, and the increasingly frequent need to meet costs and nutritional quality, some tools, in addition to the software already available, could help them to streamline their work, ensuring the offer of nutritionally balanced menus, with low cost and frequency of preparation repetitions, reducing waste and assisting the institutional foodservices in gaining autonomy, exclusivity, and sustainability.

### Literature Review

Studies conducted in the last two decades [12–14, 16–19] demonstrated that, after the implementation of the WFP in 1976, the meals offered in different institutional foodservices were inadequate compared with the nutritional parameters established [4, 6], with excessive energy supply in a single meal. Some authors also demonstrated that the workers of the companies registered with the WFP presented an increase in body mass index (BMI) and prevalence of overweight [18, 19]. The results of these studies indicate that inadequacies regarding daily energy intake and macronutrients can promote worsening quality of life, weight gain, and, consequently, an increase in chronic non-communicable diseases of workers who eat in institutional foodservices [20]. In addition, sodium and lipids are often above the requirements of the WFP [21].

When analyzing the energy density of food and beverages served in 21 companies registered in the Program, in the city of São Paulo, a positive correlation was observed between energy density and the supply of protein, total fat, and fiber, evidencing the importance of improving the quality of meals offered to diners [22]. Thus, even when the company is registered with the WFP and is supervised by a nutritionist, recommendations diverge [23]. More recently, in 2020, a group of researchers from Northeastern Brazil observed that although leafy greens, fruits, and natural beverages are supplied daily, so are ultra-processed products, mainly sweets and artificial beverages, in forty institutional foodservices analyzed [15]. This evidence demonstrates the need for corrective actions for menu planning [22, 23].

Given the context of a menu that disregards WFP recommendations, we raise the question: is there any tool to help the nutritionist to develop a menu that satisfies the diners from both a sensory point of view and a nutritional aspect, and that has a cost which generates profit or prevents losses? For this, one possibility is using a mathematical model that allows the automated generation of monthly menus of meals to be offered in the institutional foodservice, meeting nutritional requirements, and ensuring that the food is healthy, low cost, and adequate to the consumer’s palate.

The hypothesis of creating menus through computational tools began in 1945, when Stigler proposed a heuristic to find a diet with a minimum cost that satisfied nutritional recommendations [24]. Later, with the advent of the Simplex Method, Dantzig solved this problem exactly [25]. Since then, the use of mathematical tools for menu generation has evolved, with several proposed models that make possible the achievement of different objectives [26, 27].

Recent studies have demonstrated the wide application of linear programming and mixed integer programming to design diets optimized for various aspects, from health to menu cost [28–35].

The high flexibility of mathematical modeling allows its application for various purposes. Soden and Fletcher used it to build diets based on the dietary preferences of individuals and the general guidelines of healthy eating [36]. In 2006, Darmon applied this tool to predict the impact of cost constraint on food choices necessary to provide a nutritionally adequate diet for French women [37]. Linear programming was also used in studies to create dietary plans that met the main dietary recommendations for cancer prevention [29, 38] and for achieving nutrient recommendations for pregnant women in Malaysia [31]. Linear programming based mathematical models were proposed at both the individual level and for populations. In 2015, Okubo explored optimal dietary intake patterns to achieve nutritional goals for Japanese adults [11], and, in this same populational scope, Verly-Jr assessed how much nutrient content can be increased by means of a modeled diet without cost increase for low-income Brazilian families [32]. Linear programming has also been used in sustainability issues, where a nutritionally adequate and accessible school lunch menu with greenhouse gas reduction was studied and developed [34].

The most common approach to this problem is to apply linear programming models to define the amount of each food that should be ingested in a time interval, with no concern about how these foods will be distributed throughout this period [11, 29, 31, 32, 35]. However, this approach is unfit for WFP, in which the goal is a monthly menu. For this, the most appropriate approach is to use mixed integer programming, with binary variables that represent the meals served each day [33]. Table 1 provides all features considered in previous papers in comparison with the features used in the present study.

In this context, several issues beyond nutritional requirements must be considered. The spacing between repetitions of the same dish [8, 39], the palatability of each day’s combinations [40], and the eating habits of the population [11, 32] are important objectives in the preparation of menus.

**Table 1:** Features used in the modeling of the present study compared with previously published work

Author (year)	Objective	Population	Optimization strategy	Features considered in the present study									
				Energy	Macronutrients	Fibers	Color monotony	Repetition constraint	Spacing	Portion size	Cost	Eating habits	Daily menus
Soden & Fletcher (1992)	Generate individually acceptable and nutritionally adequate diets using linear programming	Overweight adult women (50 years old)	Linear Programming	✓	✓	✓				✓		✓	
Darmon et al. (2006)	Evaluate the impact of a cost constraint on the food choices needed to provide a nutritionally adequate diet	Adult french women	Linear Programming	✓	✓	✓				✓	✓	✓	
Masset et al. (2009)	Use linear programming to generate eating plans that meet the 2007 major dietary recommendations issued by the World Cancer Research Fund/American Institute of Cancer Research	Men and women of a Public University	Linear Programming	✓	✓	✓				✓		✓	
Okubo et al. (2015)	Explore optimal food intake patterns according to the nutrient recommendations of the Dietary Reference Intakes (DRIs) while incorporating typical Japanese food selections	Healthy Japanese adults (31–76 years old)	Linear Programming	✓	✓	✓				✓		✓	
Alaini et al. (2019)	Build a balanced, tasty, accessible, and low-cost diet that helps prevent cancer	Healthy Adults from a Public University in Kuala Lumpur	Linear Programming	✓	✓	✓				✓	✓		
Hamid et al. (2019)	Check if the recommended nutrient intake for pregnant women could be formulated from locally available foods in Malaysia.	Pregnant women	Linear Programming	✓	✓	✓				✓	✓		
Verly-jr et al. (2019)	Evaluate how much the nutrient content can be increased through a modeled diet, without increasing the cost, for low-income Brazilian families.	Low-income healthy Brazilian adults	Linear Programming	✓	✓	✓				✓	✓	✓	
Benvenuti & De Santis (2020)	Develop a new programming methodology to enhance a deal diet with the acceptability of meal plans	Schools and seniors	Binary Linear Programming	✓	✓	✓		✓		✓	✓	✓	✓
**Current study**	Propose a mathematical model to generate a one-month menu that meets the nutritional recommendations of the Workers’ Food Program (WFP), based on typical Brazilian culinary dishes with cost minimization.	Brazilian adults – workers in general	Mixed Integer Linear Programming	✓	✓	✓	✓	✓	✓	✓	✓	✓	✓

According to the data described in the Table 1, we can observe that the features “color monotony” and “spacing” were attributes specially considered in the present work. In addition, the features “repetition constraint” and “daily menus”, which were included in our model, were only considered in one study (Benvenuti & De Santis, 2020). It is important to note that the “daily menu” column refers to complete menu served in a day (i.e., lunch), unlike other studies that use mathematical models to generate daily diets. For instance, the present study planned a daily menu that meets the World Food Program (WFP) recommendations for Brazilian workers adults, rather than multiple meals prescribed to enhance total of calories and nutrients according to daily recommendations, as is done in other studies and may be referred to as a “daily diet”.

As far as we know, no comparable studies have been conducted in Brazil, especially with institutional foodservice menus. Thus, the current article proposes a mathematical model to generate nutritionally ideal food intake patterns that meet national recommendations based on typical Brazilian culinary dishes [4, 6] with cost minimization.

## Methods

### Description

In this project, a mixed integer programming model was built to formulate daily menus for a Hospital foodservice over a period of one month, using binary variables to indicate whether each preparation is present on each day. The mathematical model formulation is fully disclosed in supplementary material 1. The month was considered as four weeks, composed of five days each. The main goal was to minimize overall costs while respecting the nutritional requirements and eating habits of the population.

The meals (lunch or dinner) that make up the daily menus should necessarily contain the following preparations and their servings (in grams):

- White rice: 120 g;
- Beans (pulses from the Fabaceae family): 90 g;
- Base dish option (rice and beans): 90 to 120 g;
- Protein dish: 80 to 110 g;
- Protein dish option (omelet): 110 g;
- Salads (two options): 20 g for raw and 80 g for cooked vegetables;
- Side dishes: 80 to 130 g;
- Dessert (one option).

The divisions were stipulated based on the energy and macronutrient distribution reference according to the recommendations of the WFP, which will be detailed below, and based on the data on servings (in grams) for healthy adult employees of an institutional foodservice located in a public hospital. The quantities used as a reference in this study are in accordance with the characteristics of the diners (weight, age, energy expenditure), and were based on the actual servings of the institutional foodservice used as a model; they were also in accordance with estimates of the divisions according to the recommendations of the WFP [6].

After defining the size of the servings, a database was elaborated with 205 culinary dishes. Of these 205 culinary dishes, 4 were rice; 3 beans; 47 side dishes; 54 protein dishes; 8 omelets; 40 salads (26 leafy greens and 14 others), and 47 sweet desserts (of which 6 were fruits). The culinary dishes that comprised the general list, used as the basis for choosing weekly menus, were selected based on the frequency criterion, that is, preparations that are usually served in the institutional foodservice in the Southeast region of the country, using the menu served to the employees of a medium-sized hospital institutional foodservice as a reference (501 to 2000 meals/day) [41].

All culinary dishes (n = 205) were detailed regarding the list of ingredients (1,285 ingredients) used. The researchers had access to the standard recipes used in the hospital’s institutional foodservice. From the details of the ingredients and their respective quantities, the amount of energy and nutrients (proteins, total fats, saturated fats, carbohydrates, and total dietary fiber) was calculated based on data from food composition tables: the Food Composition Table (TACO) [42] and the Table of the Nutritional Composition of Foods Consumed in Brazil [43]. The price of each dish and its respective serving were also calculated. The prices of the ingredients that comprised each of the culinary preparations served were obtained from a local federal public supply company.

The culinary preparations were also analyzed in their entirety for sensory aspects, such as color and texture. We classified all dishes according to the physical-chemical and sensory aspects and textures, considering soft foods (examples: cauliflower gratin, stroganoff, soufflé, lasagna); rich in sulfur (examples: those containing broccoli, cauliflower, cabbage, sweet potatoes, and turnips as their main ingredient or in their composition); with sauce (examples: spinach in white sauce, lasagna, pasta, meat with sauces); and fried foods (examples: all preparations that were arranged using oil immersion). The dishes were also classified according to the color in the final dishes (examples: brown for red meats; green for leafy salads; red for preparations with tomato sauce; yellow for preparations with potato, corn, cheese, and for fried preparations; colored for preparations with more than two colors, which had no predominant color; orange for preparations with carrot, pumpkin, and papaya; white for preparations with cooked cauliflower, onion salad, coconut, cake, blancmange, *canjica* [a Brazilian recipe composed of white corn, milk, and cinnamon], turnip, white cabbage and rice; beige for preparations with chicken and/or breaded recipes).

### Nutritional requirements

Nutritional constraints were based on the requirements proposed by the WFP [6] which recommends the following parameters for main meals such as lunch and/or dinner:

- 600 Kcal ≤ Energy ≤ 800 Kcal;
- Carbohydrates: from 55 to 75% of the daily energy recommendation, being 60% in the lunch meal;
- Proteins: 10–15% of daily energy recommendation, being 15% in the lunch meal;
- Total fats: 15–30% of daily energy recommendation, being 25% in the lunch meal;
- Total fibers: > 25 g/day, being 7 to 10 g in the lunch meal.

Regarding the upper limits used for carbohydrates and proteins, we emphasize that for carbohydrates, the recommendation values could not exceed 65% of daily energy recommendation, and for proteins the interval between 15% and 20% of daily energy recommendation was considered. This is because some international recommendations have different macronutrient distribution ranges from those stipulated by the WFP. As an example, we cite the recommendations of the Institute of Medicine [1] of 45 to 65% carbohydrates, 10 to 35% proteins, and 20 to 35% total fat. These adjustments were necessary because, in Brazil, the combination of “base dish”, represented by rice and beans, associated with protein dishes exceeds, in most cases, the 15% protein limit in lunch meals [44].

Note that the amount of sodium present in the menus was not considered as a constraint in this study. This is because we considered the miscalculation present in national nutritional tables, since the exact amount of salt or sodium-containing condiments used in culinary dishes is often difficult to measure and/or standardize.

The WFP recommendations also allow an increase of 20%, equivalent to 400 kcal, in relation to the daily energy recommendation of 2,000 kcal that can reach 2,400 kcal in the daily energy recommendation. Thus, a main meal can provide up to 960 kcal when using the upper margin of 800 kcal recommended. This increase is mainly predicted for workers who have high energy expenditure in their work activities and, therefore, require greater energy and nutrient intake. However, considering that a meal with 800 kcal or more could, in the long term, increase energy consumption and the risks of associated diseases [6] the 20% increase allowed by the WFP was not considered as a criterion for the present study.

### Additional considerations

#### “Base dish”

Once each week a different type of rice and a different type of beans/pulses should be offered, that can be chosen instead of white rice and traditional beans. These two should not coincide and should not be offered on the same day as a pasta-based preparation.

#### “Protein dish”

A single protein dish option, based on red or white meat, or omelet — served as an option to the protein dish — should be offered.

#### “Salads”

Two salad options should be offered, being a leafy green option and a raw or cooked option. The diner can only choose one of the options served and the nutritional constraints are computed separately.

#### “Dessert”

To stimulate the healthy habits of diners, fruits should be offered three times a week, on non-consecutive days (Monday, Wednesday, and Friday). On the remaining days any kind of sweet dessert can be offered.

#### “Fatty meats, sausages, and fried foods”

Aiming at the health of the diner, pork meat was limited to twice a month, sausages to once a month, and fried foods to twice a month.

#### “Composition”

For this menu, a day should have at most one dish based on wheat flour (e.g., pancake, pasta, pie) and one dish containing a sulfur-rich ingredient. Moreover, considering texture, a dish with a “soft” texture should not be combined with another containing sauce.

#### “Color monotony”

No more than three dishes should have the same color on the same day. The color of the two salads and the side dish should also be different from each other.

#### “Eating habits”

To adapt the menu to Brazilian food habits, every day on which stroganoff is served the side dish should be shoestring potatoes. Moreover, due to the high energy concentration of shoestring potatoes, the value of nutritional constraints on the days when this is served may be extrapolated, thus, the number of times this dish is served in the month is limited.

#### “Spacing”

The dishes cannot be repeated frequently, since this decreases the satisfaction of the diners with the menu [39]; this was a difficulty for the modeling technique used. One way to solve this problem is to limit the repetition of dishes in each time interval, as done in the work by Benvenuti and De Santis [33]. However, due to the planning horizon and the constraints imposed, completely avoiding repetitions is impossible and limiting the maximum number of repetitions does not guarantee an appropriate time interval between the consecutive times the meal is served.

To solve this problem, we used a series of soft constraints with weights in the objective function to make the spacing adequate and consistent with the eating habits of Brazilians. The priority of non-repetition is for the protein dishes since they are the main variation between the menus. Therefore, the occurrence of each protein dish was limited to once in the planning horizon. Moreover, multiple beef dishes, or poultry dishes, could not occur on consecutive days. This repetition was penalized in the objective function.

Four-time intervals (3 days, one week, two weeks, and one month) were also defined, within which repetitions should be avoided, in decreasing order of priority. Thus, repetitions of preparations in these intervals were penalized in the objective function. The weight assigned to them increases with the number of repetitions, to make it clear in the model that repeating two different preparations once each is better than repeating the same preparation twice.

Finally, similar preparations should not be offered in short time intervals. To prevent this, several categories were constructed based on the eating habits of Brazilians (for example, dishes with potatoes, pancakes) so that similar preparations could not be offered on consecutive days and their weekly repetition was penalized in the objective function.

## Results

The supplementary material 2 shows the weekly menus obtained considering a planning horizon of four weeks. In it, the “base dishes” (rice and beans) were omitted, as they are present every day. Despite the outputs generated with the combinations of culinary dishes, the person in charge, such as the nutritionist, may make small adjustments if they deem it necessary, for example, on the 13th, when *feijoada* (a typical Brazilian recipe composed of black beans, sausage, salted meat, and bacon) is served, the side dish can be changed to *farofa* (cassava flour).

The average daily nutritional values and costs of each week menu is shown in Table 2. For carbohydrates, proteins, and total fats, the fraction of the total energy that each of these nutrients represents on average is also provided for comparison with WFP requirements.

**Table 2:** Nutritional values and weekly menu cost (n = 20 days) using average meal values. Hospital Institutional Food Service, Campinas, São Paulo, Brazil (2019)

Week	Cost -US$ (R$)	Energy (kcal)	Carbohydrates (kcal)	Proteins (kcal)	Total Fats (kcal)	Saturated Fats (kcal)	Fibers (g)
**1**	74,9 (410.51)	724.49	422.98	131.39	183.97	59.44	15.03
**2**	66,27 (363.2)	719.83	416.22	129.78	187.41	72.65	26.61
**3**	74,08 (406.03)	703.75	416.35	123.18	163.71	41.46	16.8
**4**	71,45 (391.6)	719.82	415.22	128.75	178.36	46.13	14.98
**Mean**	**71,68 (392.84)**	**716.97**	**417.69**	**128.28**	**178.36**	**54.92**	**18.35**
**(% of total energy)**	-	-	**58.26**	**17.89**	**24.88**	-	-

**Fig. 1:**
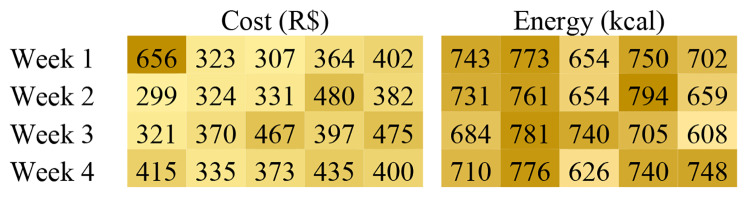
Daily average meal energy and menu cost values (n = 20 days). Hospital Institutional Food Service, Campinas, São Paulo, Brazil (2019)

**Fig. 2:**
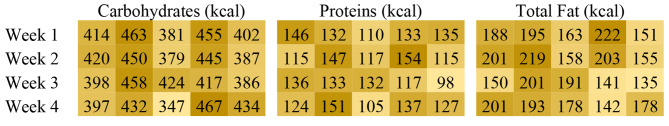
Daily average meal macronutrients (carbohydrates, proteins and total fat) values (n = 20 days). Hospital Institutional Food Service, Campinas, São Paulo, Brazil (2019)

To better illustrate the data, Figs. 1 and 2 present heatmaps with the average daily cost and nutrients values. Each row represents a week, and each cell represents a day. Lighter cells represent smaller values and darker cells indicate higher values. Since there are two salad options in each day, the nutritional values are different for each choice. In these figures, the values represent the average between the two options (Figs. 1 and 2).

**Fig. 3:**
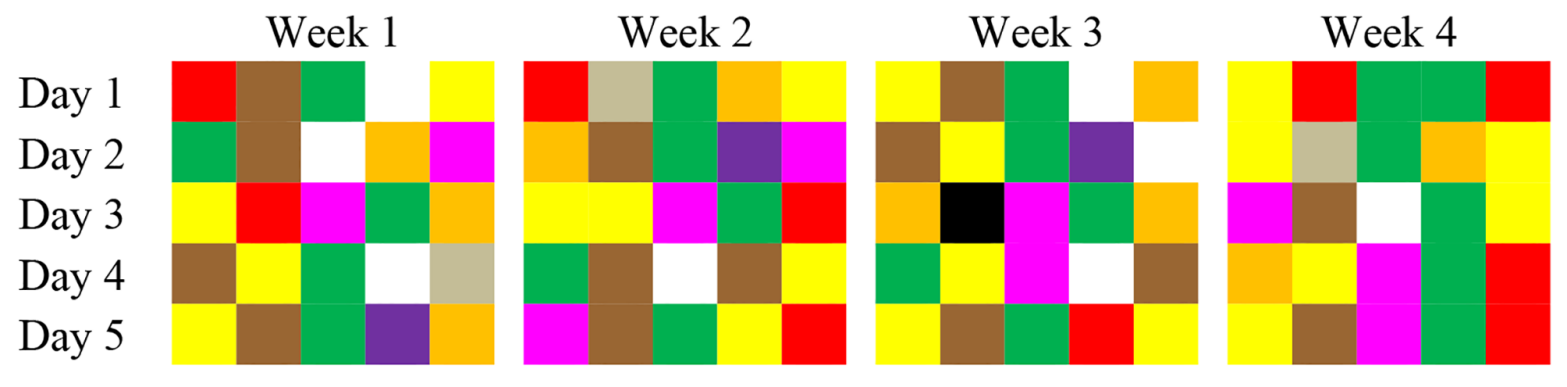
Meals color distribution for one month menu (n = 20 days). Hospital Institucional Food Service, Campinas, São Paulo, Brazil (2019)

The solution obtained allows us to conclude that, in fact, the constraints used were sufficient to ensure that the combinations were palatable from the point of view of Brazilian eating habits, and to ensure a month of healthy and nutritionally balanced meals. We also achieved the desired spacing. No week presented a repetition of dishes or the presence of two dishes considered similar. There were also few repetitions in the two-week period. Repetitions of the same dishes throughout the planning horizon were also scarce and, of those that were repeated, the majority were omelets or fruits, for which less variety is available. Bovine protein dishes only occurred on two pairs of consecutive days and, for poultry, this never occurred.

Regarding meeting nutritional requirements, the menu was adequate within the range recommended by the WFP [6]. It is possible to note that the mean energy (Table 2), according to the established servings, met the recommendations, being 716.97 kcal/meal (std = 61.4 kcal/meal). Similarly, adequate values of carbohydrates, proteins, and lipids were achieved, expressed in kcal per meal. The four-week menu offered, on average, 417.19 kcal/meal (std = 36.7) of carbohydrates, 128.28 kcal/meal (std = 15.9) of proteins, and 178.36 kcal/meal (std = 27.6) of lipids, corresponding to an average of 58% of carbohydrates, 18% protein, and 25% lipids in the meal. Furthermore, Figs. 1 and 2 show the daily values of each macronutrient, indicating their fluctuation over the planning horizon. It can be observed that the nutritional values have much smaller fluctuations than the cost, for instance, since the menu goal is to have daily nutritionally balanced meals, while minimizing overall cost, not daily cost. Note that studies conducted over five years (from 2006 to 2011) showed an imbalance between the nutrients of the menus of institutional foodservices registered in the WFP, especially exceeding the amount of total energy, as shown by Barbosa et al. [44]. The percentage value of proteins obtained in this model is above the maximum WPF recommendation (15%) but does not exceed the IOM recommendation of 35% [1]. However, adjustments can be made by reducing the main/protein dish serving in the institutional foodservice itself. Moreover, the combination of the menu did not exceed the percentage of lipids, or the total amount of energy of the lunch, which is an important premise, in order to provide workers with a balanced meal, avoiding the onset of diseases such as obesity and dyslipidemias [18, 19, 44].

The combinations of the preparations were also adequate, since all culinary dishes and combinations generated (for example, protein dish and side dish) are in line with Brazilian eating habits. Since the variety of colors of the food served was also considered, minimizing the monotony of colors and the combinations of the preparations served on the same day was possible. Figure 3 illustrates how these colors are distributed by day, indicating that there were low color repetitions in the same day and menus were colorful, with no monotony during the month. Therefore, we did not need to add or exclude categories from the menu, which continued with the following options: starter or salad, “base dish” (rice and beans), side dish, protein dish, and dessert. Regarding dessert, despite the recommendations for serving fruit daily, the weekly menus presented sweet desserts on alternate days, since the institutional foodservice used as a model served this option in addition to the fruit on alternate days. However, the fruits were not counted and/or presented on the same day as a sweet dessert, since the diner is required to select one of the options.

## Conclusion

The results of the current study enable us to conclude that the developed model achieves the objective of generating appropriate menus for the reality of the institutional foodservice and Brazilian eating habits, besides ensuring adequate spacing between repetitions of culinary dishes and cost minimization. Moreover, the tool used is very flexible and can be easily adapted to specific realities, with the addition and removal of constraints.

The model presented represents an advance in the modeling techniques commonly used to solve problems involving human diet, because it allows an approach focused on the generation of menus concerned with nutritional and cultural issues, besides ensuring a palatable diet with few repetitions and color variety. This perspective is unusual in the models found in the literature, which are more focused on levels of food consumption in a planning horizon, without worrying about the way these foods will be offered and distributed during the period.

This model could also help in the dynamics of elaborating menu meals in the institutional foodservice, generating an output that encompasses different aspects, that is, considering nutrient composition, meeting the recommendations of the WFP, and the issue of important sensory aspects in the quality of the menus and their acceptability such as color and texture combinations. The model can also meet the specific demands of each location, such as the offer of specific dishes, spacings, and types of constraints, among others, generating combinations with a lower cost. Note that adjustments may be necessary for the output to meet all the needs of each institutional foodservice.

## Electronic supplementary material

Below is the link to the electronic supplementary material.

Supplementary Material 1

Supplementary Material 2

## Data Availability

The datasets used and/or analysed during the current study are available from the corresponding author on reasonable request.
