# Supplementary material for: Optimized menu formulation to enhance nutritional goals: design of a mixed integer programming model for the workers’ food program in Brazil
Source: BMC Nutr. 2023 Mar 20;9:51. doi: 10.1186/s40795-023-00705-0 (PMC10026400; doi:10.1186/s40795-023-00705-0)
Supplement: Supplementary file 1 — Supplementary Material 1 [file 40795_2023_705_MOESM1_ESM.docx]

**Supplementary Material. Mathematical model formulation**.

**Menu Optimization Model**

We provide the MIP used for menu optimization. The mathematical notation is based on the following.

**Sets**

- *I*: Set of preparations. In order to make notation simpler, we will assume that *i* = 1 represents stroganoff and *i* = 2 represents shoestring potatoes;
- *S* ⊂ *I*: Set of salads;
- *T*: Set of days in the planning horizon (T = {1, 2,..., 20});
- *W*: Set of weeks (W = {1, 2, 3, 4});
- *N*: Set of nutrients (N = {1, 2, 3, 4, 5}). The nutrients that these numbers represent are, respectively, total energy amount, carbohydrates, proteins, total fats and fiber;
- *P*: Set of categories of similar preparations (e.g., dishes with potatoes, pancakes);
- *C*: Set of colors;
- *V* ⊂ *I*: Set of preparations that are not considered in nutritional constraints (e.g., omelets, base dish option, salads);
- *K*: Set of characteristics of preparations that have a limit in the planning horizon (e.g., fried foods);
- *Z*: Set of characteristics of preparations (e.g., “soft” preparations, leafy green salads). Only one preparation with characteristic *z* can be served on each day, *z* $\in$ *Z*. It is clear that *Z* ≠ *K*;
- *H*: Set of preparation types (e.g., white rice, beans, protein dish, side dish).

**Parameters**

- $c_{i}$: Cost of preparation *i*, *i* $\in$ *I*;
- $e_{i,n}$: Amount of nutrient *n* in preparation *i*, *n* $\in$ *N*, *i* $\in$ *I*;
- $l_{n}$: Lower bound of nutrient *n*, *n* $\in$ *N*;
- $u_{n}$: Upper bound of nutrient *n*, *n* $\in$ *N*;
- $\alpha_{h}$: Daily number of type *h* preparations, *h* $\in$ *H*;
- $\beta_{k}$: Maximum number of preparations sharing characteristic *k* that can be served over the planning horizon, *k* $\in$ *K*;
- $M_{1}$: Parameter used to minimize repetitions (we adopted $M_{1}$ = 100);
- $M_{2}$: Parameter used in nutrition constraints (we adopted $M_{2}$ = 1000).

**Variables**

- $x_{i,t}$: Binary variable that indicates whether preparation *i* is served on day *t*, *i* $\in$ *I*, *t* $\in$ *T*;
- $\tau_{n,s,t}$: Total amount of nutrient *n* in the meal served in day *t* considering *s* as salad option, *n* $\in$ *N*, *s* $\in$ *S*, *t* $\in$ *T*. This variable is not necessary in the model, but is presented to simplify notation;
- $d_{i,t,j}$: Binary variable used to minimize repetitions in three-day intervals, *i* $\in$ *I*, *t* $\in$ *T*, *j* $\in$ {1, 2};
- $f_{i,w,j}$: Binary variable used to minimize repetitions in the same week, *i* $\in$ *I*, *w* $\in$ *W*, *j* $\in$ {1, 2,..., 5};
- $q_{i,w,j}$: Binary variable used to minimize repetitions in consecutive weeks, *i* $\in$ *I*, *w* $\in$ *W*, *j* $\in$ {1, 2,..., 10};
- $r_{i,t}:$ Binary variable used to minimize repetitions in the overall planning horizon, *I* $\in$ *I*, *t* $\in$ *T*;
- $a_{t}$: Continuous variable used to avoid serving poultry dishes in consecutive days, *t* $\in$ *T*;
- $b_{t}$: Continuous variable used to avoid serving beef dishes in consecutive days, *t* $\in$ *T*;
- $g_{p,w}$: Continuous variable used to minimize repetitions of similar preparations in a week, *p* $\in$ *P*, *w* $\in$ *W*;

The model used to solve the problem presented and described in the paper is given by Equations (A.1) to (A.36).

| Min | $\sum_{i\in I} \sum_{t\in T} x_{i,t}c_{i}+50M_{1}\sum_{i\in I} \sum_{t\in T} \sum_{j=1}^{2} j*d_{i,t,j}+25M_{1}\sum_{p\in P} \sum_{w\in W} g_{p,w}+25M_{1}\sum_{i\in I} \sum_{w\in W} \sum_{j=1}^{5} j*f_{i,w,j}+5M_{1}\sum_{i\in I} \sum_{w\in W} \sum_{j=1}^{10} j*q_{i,w,j}+M_{1}\sum_{i\in I} \sum_{t\in T} t*r_{i,t}+3M_{1}\sum_{t\in T} \left( a_{t}+b_{t} \right)$ | (A.1) |
| --- | --- | --- |
| s.t. | $\tau_{n,s,t}=x_{s,t}e_{s,n}+\sum_{i\in I\backslash V} x_{i,t}e_{i,n}, \forall n\in N, s\in S, t\in T$ | (A.2) |
|  | $\tau_{n,s,t}\geq l_{n}-M_{2}\left( 1-x_{s,t} \right),\forall n\in\{1,5\}, s\in S,t\in T$ | (A.3) |
|  | $\tau_{n,s,t}\geq l_{n}\tau_{1,s,t}-M_{2}\left( 1-x_{s,t} \right),\forall n\in\{2,3,4\},s\in S,t\in T$ | (A.4) |
|  | $\tau_{1,s,t}\leq u_{1}+M_{2}x_{2,t},\forall s\in S, t\in T$ | (A.5) |
|  | $\tau_{n,s,t}\leq u_{n}\tau_{1,s,t}+M_{2}x_{2,t},\forall n\in\{2,3,4\},s\in S,t\in T$ | (A.6) |
|  | $\sum_{i\in h} x_{i,t}=\alpha_{h},\forall h\in H,t\in T$ | (A.7) |
|  | $\sum_{i\in\{\mathrm{sweet}\}} x_{i,\left( w-1 \right)*5+2}=1,\forall w\in W$ | (A.8) |
|  | $\sum_{i\in\{sweet\}} x_{i,\left( w-1 \right)*5+4}=1,\forall w\in W$ | (A.9) |
|  | $\sum_{i\in\{sweet\}} \sum_{t\in T} x_{i,t}=8$ | (A.10) |
|  | $\sum_{i\in\{rice option\}} \sum_{t=\left( w-1 \right)*5+1}^{w*5} x_{i,t}=1,\forall w\in W$ | (A.11) |
|  | $\sum_{i\in\{beans option\}} \sum_{t=\left( w-1 \right)*5+1}^{w*5} x_{i,t}=1,\forall w\in W$ | (A.12) |
|  | $\sum_{i\in\{base dish option\}} x_{i,t}+\sum_{i\in\{\mathrm{pasta}\}} x_{i,t}\leq1,\forall t\in T$ | (A.13) |
|  | $\sum_{i\in k} \sum_{t\in T} x_{i,t}\leq\beta_{k},\forall k\in K$ | (A.14) |
|  | $x_{2,t}=x_{1,t},\forall t\in T$ | (A.15) |
|  | $\sum_{t\in T} x_{2,t}\leq2$ | (A.16) |
|  | $\sum_{i\in z} x_{i,t}\leq1,\forall z\in Z,t\in T$ | (A.17) |
|  | $\sum_{i\in c} x_{i,t}\leq3,\forall t\in T,c\in C$ | (A.18) |
|  | $\sum_{i\in\left( S\cup\{side dish\} \right)\cap c} x_{i,t}\leq1,\forall t\in T,c\in C$ | (A.19) |
|  | $\sum_{i\in p} \left( x_{i,t}+x_{i,t+1} \right)\leq1,\forall p\in P,t\in T\backslash\{20\}$ | (A.20) |
|  | $\sum_{t\in T} x_{i,t}\leq1,\forall i\in\{protein dish\}$ | (A.21) |
|  | $x_{i,t}+x_{i,t+1}+x_{i,t+2}\leq1+d_{i,t,1}+d_{i,t,2},\forall i\in I,t\in T\backslash\{19,20\}$ | (A.22) |
|  | $\sum_{j=1}^{5} x_{i,5\left( w-1 \right)+j}\leq1+\sum_{j=1}^{5} f_{i,w,j},\forall i\in I,w\in W$ | (A.23) |
|  | $\sum_{j=1}^{10} x_{i,5\left( w-1 \right)+j}\leq1+\sum_{j=1}^{10} q_{i,w,j},\forall i\in I,w\in W\backslash\{4\}$ | (A.24) |
|  | $\sum_{t=1}^{5} x_{i,t}+\sum_{t=16}^{20} x_{i,j}\leq1+\sum_{j=1}^{10} q_{i,4,j},\forall i\in I$ | (A.25) |
|  | $\sum_{t\in T} x_{i,t}\leq1+\sum_{t\in T} r_{i,t},\forall i\in I$ | (A.26) |
|  | $\sum_{i\in\{beef\}} \left( x_{i,t}+x_{i,t+1} \right)\leq1+b_{t},\forall t\in T\backslash\{20\}$ | (A.27) |
|  | $\sum_{i\in\{poultry\}} \left( x_{i,t}+x_{i,t+1} \right)\leq1+a_{t},\forall t\in T\backslash\{20\}$ | (A.28) |
|  | $\sum_{i\in p} \sum_{j=1}^{5} x_{i,5\left( w-1 \right)+j}\leq1+g_{p,w},\forall p\in P,w\in W$ | (A.29) |
|  | $x_{i,t},r_{i,t}\in\{0,1\},\forall i\in I,t\in T$ | (A.30) |
|  | $\tau_{n,s,t}\in R_{+},\forall n\in N,s\in S,t\in T$ | (A.31) |
|  | $d_{i,t,j}\in\{0,1\},\forall i\in I,t\in T,j\in\{1,2\}$ | (A.32) |
|  | $f_{i,w,j}\in\{0,1\},\forall i\in I,w\in W,j\in\{1,2,\ldots,5\}$ | (A.33) |
|  | $q_{i,w,j}\in\{0,1\},\forall i\in I,w\in W,j\in\{1,2,\ldots,10\}$ | (A.34) |
|  | $a_{t},b_{t}\in R_{+},\forall t\in T$ | (A.35) |
|  | $g_{p,w}\in R_{+},\forall p\in P,w\in W$ | (A.36) |

The Objective Function (A.1) minimizes the overall cost and controls soft constraints. The penalizing weights used were presented. However, it may be interesting to fine tune them in other applications. They are in decreasing priority order to represent our preferences of avoiding preparation repetitions within short time intervals. Moreover, some summations include a *j* factor, so repeating many preparations once is better than repeating the same preparation many times. Constraint (A.2) defines the total amount of each nutrient for each day considering a salad option. Constraints (A.3) to (A.6) control the nutritional requirements. As stated in the Section 2, the lower bounds are considered only for the salads that are served and upper bounds are not considered when shoestring potatoes are served. Moreover, upper, and lower bounds for carbohydrates, proteins and total fats depend on the total energy amount. Fiber upper bound is not considered.

Constraint (A.7) ensures that the correct dishes are served each day. Constraints (A.8) to (A.10) define that sweets are served every Tuesday and Thursday. Constraints (A.11) and (A.12) state that, once a week, base dish options are served. Constraint (A.13) ensures that at most one base dish option or pasta preparation is served each day. Constraint (A.14) limits, in the planning horizon, the offer of preparations that should not be served frequently (e.g., sausages). Constraints (A.15) and (A.16) define that stroganoff and shoestring potatoes are always served together and that it can happen only twice in the planning horizon. Constraint (A.17) avoids serving preparations with similar characteristics on the same day (e.g., sulfur-rich ingredients). Constraints (A.18) and (A.19) regulate color monotony and Constraints (A.20) to (A.29) control spacing, as described in the Methodology section. Finally, Constraints (A.30) to (A.36) define variables types and limits.
