# Supplementary material for: Optimized menu formulation to enhance nutritional goals: design of a mixed integer programming model for the workers’ food program in Brazil
Source: BMC Nutr. 2023 Mar 20;9:51. doi: 10.1186/s40795-023-00705-0 (PMC10026400; doi:10.1186/s40795-023-00705-0)
Supplement: Supplementary file 2 — Supplementary Material 2 [file 40795_2023_705_MOESM2_ESM.docx]

Supplementary Table

Title: Weekly menus obtained considering a planning horizon of four weeks.

| **Weeks** | **Days** | **Salad - option 1** | **Salad - option 2** | **Protein dish** | **Protein dish option** | **Garnish** | **Dessert** | **Base dish option** |
| --- | --- | --- | --- | --- | --- | --- | --- | --- |
| **1** | **1** | Chicory | Onion salad | Dried meat and onions | Omelet | Bolognese polenta | Banana | "Arroz carreteiro" (white rice with dried meat)* |
|  | **2** | White cabbage | Carrot | Steak and onions | Ham, cheese, mozzarella and parsley omelet | Braised zucchini (with bacon) | Gelatin dessert with single cream |  |
|  | **3** | Cress | Pasta salad (screw pasta, green and red bell peppers and onion) | Chicken pancake with tomato sauce | Onion, tomato and parsley omelet | Bovillon potatoes (braised with margarine, garlic and ham) | Orange |  |
|  | **4** | Escarole | Turnip | "Fish à Portuguese Style" (Fish with green and red bell pepper, egg, onion, black olive, potato, carrot and sauce)* | Pea and onion omelet | Roasted eggplant | Peanuts Praline |  |
|  | **5** | Raw leafy greens (or kale) | Beetroot | "Bife Fricandolê" (steak with bacon)* | Mozzarella cheese, oregano and tomato omelet | Cassava Mash | Papaya | "Feijão tropeiro" (beans, onions, garlic, pork crackling, greens/kale and cassava flour)* |
| **2** | **6** | Broccoli | Butternut squash (or Roasted pumpkin) | Chicken croquette | Mozzarella cheese and sausage omelet | Pasta with tomato sauce | Pineapple |  |
|  | **7** | Lettuce | Grated beetroot | "Bife à Camões" (steak with egg on top)* | Mozzarella cheese and green olives omelet | "Quibebe" (winter pumpkin stew with dried meat)* | Colored gelatin dessert |  |
|  | **8** | "Tabbouleh" (bulgur wheat, cucumber, tomato, mint, onion, chopped parsley, lemon juice)* | Arugula (or Rocket) | Chicken meatballs | Pea and onion omelet | Mash potatoes | Watermelon | Rice with broccoli |
|  | **9** | Boiled cauliflower | Eggplant | Goulash (meat and vegetables stew) | Sweet corn, pea and tomato omelet | Sweet corn Stew | Brazilian carrot cake |  |
|  | **10** | Chard | Sweet potato | "Virado à paulista" (pork chop, tuscan sausage and egg)* | Onion, tomato and parsley omelet | Vegetables couscous | Apple | "Tutu de feijão" (beans, flocked cassava flour, flocked cornmeal, eggs, smoked sausage, onion and bacon)* |
| **3** | **11** | Chicory | Onion salad | Roast beef | Ham, mozzarella cheese and parsley omelet | Cassava Mash | Papaya |  |
|  | **12** | Cress | Beetroot | Chicken fillet with cheese sauce | Mozzarella cheese, oregano and tomato omelet | Roasted eggplant | "Maria Mole" (gelatin, sugar and dried coconut)* |  |
|  | **13** | "Macarronese" (noodles, green and red bell peppers, sweet corn and mayonnaise)* | Raw leafy greens (or kale) | "Feijoada" (beans with beef and pork stew - leg or loin, salted ribs, dried meat, bacon, sausage and pepperoni)* | Pea and onion omelet | Sautéed carrots | Orange |  |
|  | **14** | Ceaser salad (lettuce, garlic, crotons, bacon, mustard and mayonnaise) | White cabbage | "Frango à caçadora" (chicken thigh and drumstick with white wine sauce, peppers, sweet corn, lentils)* | Omelet | Baked Panko-Breaded Zucchini | Chocolate cake | "Arroz à grega" (Rice with red bell pepper, onion, raisin, chopped parsley and carrot)* |
|  | **15** | Escarole | Tomato salad | Cubed beef stew meat with vegetables (potatoes, carrots and bell peppers) | Onion, tomato and parsley omelet | Bovillon potatoes (braised with margarine, garlic and ham) | Banana |  |
| **4** | **16** | Grain salad (lentils, soy, split peas, chickpeas) | Lettuce | Minced meat pancake with tomato sauce | Mozzarella cheese, oregano and tomato omelet | Mash potatoes | Apple |  |
|  | **17** | Broccoli | Grated carrot | Grilled chicken skewer | Corn, pea and tomato omelet | Roasted potatoes | Banana pie | "Tutu de feijão" (beans, flocked cassava flour, flocked cornmeal, eggs, smoked sausage, onion and bacon)* |
|  | **18** | Boiled cauliflower | Cucumber | Baked Kibbeh (minced beef or lamb, bulgur wheat, onion, parsley) | Mozzarella cheese and sausage omelet | Sautéed vegetables | Pineapple | Rice with broccoli |
|  | **19** | Moyashi (moyashi beansprouts, tomato and onion) | Arugula (or Rocket) | "Caussolet" (cassorele with white beans, salted ribs, pepperoni sausage, chicken breast and carrots)* | Mozzarella cheese and green olives omelet | "Quibebe" (winter pumpkin stew with dried meat)* | "Goiabada" (guava paste with single cream)* |  |
|  | **20** | Summer salad (cabbage, pineapple, apple, carrot, ham and mayonnaise) | Chard | Minced meat with potatoes | Omelet | "Spaghetti aglio e olio" (pasta with garlic and olive oil)* | Watermelon |  |

*Preparations enclosed in quotation marks (" '") are typical names of national culinary preparations in Brazil.
